# Supplementary material for: Peripheral Endocannabinoid Components and Lipid Plasma Levels in Patients with Resistant Migraine and Co-Morbid Personality and Psychological Disorders: A Cross-Sectional Study
Source: Int J Mol Sci. 2024 Feb 4;25(3):1893. doi: 10.3390/ijms25031893 (PMC10855606; doi:10.3390/ijms25031893)
Supplement: Supplementary file 1 [file ijms-25-01893-s001.zip › ijms-2822417-supplementary.pdf]

Supplementary Table S1. Patients' distribution (absolute value) across PD/wPD and MD/wMD groups.

	**PD**	**wPD**
**MD**	21 (40%)	10 (20%)
**wMD**	10 (20%)	10 (20%)

Note. PD = Personality Disorder, wPD = without Personality Disorder, MD = Mood Disorder, wMD = without Mood Disorder.
